# High-Throughput Screen of Natural Product Libraries for Hsp90 Inhibitors

**DOI:** 10.3390/biology3010101

**Published:** 2014-02-10

**Authors:** Jason Davenport, Maurie Balch, Lakshmi Galam, Antwan Girgis, Jessica Hall, Brian S. J. Blagg, Robert L. Matts

**Affiliations:** 1 Department of Biochemistry and Molecular Biology, 246 Noble Research Center, Oklahoma State University, Stillwater, OK 74078, USA; E-Mails: jason.davenport@okstate.edu (J.D.); mbalch@mail.okstate.edu (M.B.); lgalam@health.usf.edu (L.G.); 2 Department of Medicinal Chemistry, The University of Kansas, 1251 Wescoe Hall Drive, Malott 4070, Lawrence, KS 66045, USA; E-Mails: A390g100@ku.edu (A.G.); jahall27@ku.edu (J.H.); bblagg@ku.edu (B.S.J.B.)

**Keywords:** Hsp90, Hsp90 inhibitors, high-throughput screen, natural products libraries

## Abstract

Hsp90 has become the target of intensive investigation, as inhibition of its function has the ability to simultaneously incapacitate proteins that function in pathways that represent the six hallmarks of cancer. While a number of Hsp90 inhibitors have made it into clinical trials, a number of short-comings have been noted, such that the search continues for novel Hsp90 inhibitors with superior pharmacological properties. To identify new potential Hsp90 inhibitors, we have utilized a high-throughput assay based on measuring Hsp90-dependent refolding of thermally denatured luciferase to screen natural compound libraries. Over 4,000 compounds were screen with over 100 hits. Data mining of the literature indicated that 51 compounds had physiological effects that Hsp90 inhibitors also exhibit, and/or the ability to downregulate the expression levels of Hsp90-dependent proteins. Of these 51 compounds, seven were previously characterized as Hsp90 inhibitors. Four compounds, anthothecol, garcinol, piplartine, and rottlerin, were further characterized, and the ability of these compounds to inhibit the refolding of luciferase, and reduce the rate of growth of MCF7 breast cancer cells, correlated with their ability to suppress the Hsp90-dependent maturation of the heme-regulated eIF2α kinase, and deplete cultured cells of Hsp90-dependent client proteins. Thus, this screen has identified an additional 44 compounds with known beneficial pharmacological properties, but with unknown mechanisms of action as possible new inhibitors of the Hsp90 chaperone machine.

## 1. Introduction

During the past 20 years Hsp90 has emerged as a major target for the development of cancer therapeutics. The story began in 1994 with the report by Whitesell and co-workers that benzoquinone ansamycins, natural products isolated from the soil actinomycetes species *Streptomyces hygroscopicus*, were inhibitors of Hsp90 and not tyrosine kinases [1]. In 1997, the crystal structure of the Hsp90 N-terminal domain in complex with the benzoquinone ansamycin, geldanamycin was published, and later that year it was demonstrated that the geldanamycin-bindng site was responsible for the binding and hydrolysis of ATP (adenosine triphosphate) by Hsp90 [2]. Subsequently in 1998, radicicol, an antibiotic isolated from the mycoparasitic fungus *Humicola fuscoatra*, was found to bind similarly to the N-terminal domain of Hsp90 [3,4], and with the additional co-crystal structure in 1999, the road to rational drug design was paved [5].

Upon the discovery of Hsp90 inhibitors, insight into the cellular function of Hsp90 rapidly emerged. Basic research led to the identification of proteins termed “clients,” which relied upon Hsp90 not only for their activity and function, but also in many cases for their stability. Disruption of Hsp90’s function resulted in the depletion of Hsp90-dependent clients from cells either by destabilizing the client and accelerating their degradation via the ubiquitin/proteasome pathway [6], or by preventing the replenishment of mature client proteins as they turned over in the cell through the inhibition of their folding, which led to the degradation of newly synthesized clients. From these early studies it became apparent that many of the Hsp90 clients were components of signal transduction cascades, and Hsp90 became know as the “signal-transduction” chaperone [7]. Excitement over Hsp90 as a cancer therapeutic target grew when it became apparent that Hsp90-dependent clients were present in all six (now eight [8]) hallmarks of cancer, and that Hsp90 inhibitors could derail multiple cellular signaling pathways simultaneously [9].

Hsp90 inhibitors based on the benzoquinone ansamycin scaffold of geldanamycin, the resorcinol scaffold of radicicol, the purine scaffold of ATP, and other unique chemical scaffolds discovered to inhibit Hsp90, are currently being evaluated in over 80 ongoing or completed clinical trials [10]. While some of the results from these trials have been encouraging, reports of hepato-, cardio-, ocular-toxicity, peripheral neuropathy and in some cases difficult dosing schedules have tempered enthusiasm for clinical Hsp90 inhibition. However, due to Hsp90’s importance toward maintaining the viability of cancer cells, significant effort is still being dedicated to the discovery and synthesis of novel and more efficacious Hsp90 inhibitors.

Because Hsp90 inhibitors affect the function of such a diverse set of cellular pathways, the inhibitors have an unusual physiological signature. Distinctive properties of Hsp90 inhibitors include: (1) the ability to inhibit multiple, yet seemingly unrelated signal transduction pathways [9]; (2) the ability to inhibit the activities of proteins for which no rational expectation for a common binding specificity exists; (3) a high differential selectivity of the compound for cancer *versus* normal cells [11,12]; and (4) protection of cells from toxicity induced by the accumulation of protein aggregates [13,14,15] (*i.e.*, aggregates of tau or Aβ amyloid).

These distinctive properties manifested by Hsp90 inhibitors have widespread and sometimes varied effects within a cell. Depending on the conditions, the physiological manifestations of this inhibition can also vary. For example, Hsp90 is highly involved in the inflammatory response, as several mediating proteins, such as IκB Kinase (IKK) [16,17] and nitric oxide synthase [18,19], are dependent upon Hsp90 for their function. Accordingly, Hsp90 inhibitors result in the down-regulation of these proteins and display anti-inflammatory activity, which makes them prominent in traditional medicine. In the case of cancer cells, accelerated growth and cell division is maintained by Hsp90-dependent clients, as is angiogenesis [20,21]. Thus, treatment with Hsp90 inhibitors results in the slowing of cell growth, inhibition of tumor vascularization, and potentially, induction of apoptosis. Similarly, the effects of Hsp90 inhibition can be seen in other medically relevant ways, including activity against viruses [22], bacteria, fungi [23], and parasites [24], specifically the causative agent of malaria, *Plasmodium falciparum* [25].

A number of high-throughput screening (HTS) assays based on a variety of techniques have been developed to screen large chemical libraries to identify new Hsp90 inhibitors (reviewed in [26]). Screens have been developed based on the ability of compounds to: (1) inhibit Hsp90 catalyzed ATP hydrolysis; (2) competitively inhibit the binding of ligand to Hsp90’s N-terminal ATP binding domain; (3) inhibit Hsp90-mediated refolding of denatured protein (e.g., luciferase); and (4) deplete cultured cells of Hsp90 client proteins. These assays have identified a large number of potential Hsp90 inhibitors. However, only a limited number of follow-up studies have been carried out to verify the mechanism of action of these compounds and to optimize their Hsp90-inhibitory activity.

As noted above, most HTS have been carried out on large chemical libraries. Here we focus on the use of a high-throughput assay to screen natural product libraries for novel inhibitors of the Hsp90 chaperone machine. The screening is based on the ability of Hsp90 inhibitors to block the refolding of thermally denatured firefly luciferase, which is catalyzed by the Hsp90 chaperone machinery present in rabbit reticulocyte lysate [27,28,29]. It was reasoned that natural products would be a fertile territory for identification of additional Hsp90 inhibitors, as it would be reasonable to expect that evolutionary pressure would give plant or other species, which have acquired pathways leading to the synthesis of secondary metabolites that inhibit Hsp90 a competitive advantage, because such compounds would be expected to inhibit the growth and development of insect and pathological pests. Furthermore, as noted in a recent review, the majority of drugs approved for use by the FDA during the past 30 to 50 years are natural products or derivatives thereof [30,31]. In addition, there is vast literature on active compounds that have been isolated from traditional folk medicines that allowed us to mine the literature for compounds identified in our screen that have properties of Hsp90 inhibitors that were discussed above.

## 2. Experimental Section

### 2.1. High-Throughput Screen of Natural Product Libraries

#### 2.1.1. Rabbit Reticulocyte Lysate

Rabbit reticulocyte lysate, prepared by lysing one volume of packed reticulocytes in two volumes of deionized water, followed by centrifugation for twenty minutes at 15,000 × g, was purchased from Green Hectares (Oregon, WI, USA).

#### 2.1.2. Denatured Luciferase

Recombinant luciferase from Promega was diluted to 0.5 mg/mL in buffer consisting of 25 mM Tricine–HCl (pH 7.8), 8 mM MgSO_4_, 0.1 mM EDTA, and 10 mg/mL acetylated BSA. Next, the solution was adjusted to include 10% glycerol and 1% Triton X-100. Finally, the luciferase solution was heated to ~41°. Once the activity of the luciferase reached ~1% of its initial value, the mixture was placed on ice, or flash frozen in liquid nitrogen and placed at −80° for storage.

To prepare the denatured luciferase for use in re-folding assays, 125 µL of the 0.5 mg/mL mixture was added into a 10 mL mixture containing 80 mM Tris HCl, pH 7.7, 8 mM Mg(OAc)_2_, 300 mM KCl, 12 mM ATP, and 20 mM creatine phosphate, and 0.8 mg/mL creatine phosphokinase.

#### 2.1.3. Assay Buffer

The assay buffer, which contains the luciferase substrate luciferin, consisted of 75 mM Tricine-HCl, pH 7.8, 24 mM MgSO4, 300 µM EDTA, 2 mM DTT, 313 µM D-luciferin, 640 µM coenzyme A, 660 µM ATP, 150 mM KCl, 10% (*v*/*v*) Triton X-100, 20% (*v*/*v*) glycerol, and 3.5% DMSO.

#### 2.1.4. Compounds Library Screen

The natural product libraries were purchased from the following companies: Microsource Spectrum Collection (University of Kansas High Throughput Screening Center, 720 compounds); TimTec (480 compounds); AnalytiCon Discovery(2,511 compounds); BioFocus (272 compounds); and; BioMol Life Sciences/ ENZO Life Sciences (596 compounds). Of the 2,608 compounds that were present in the Microsource, TimTec, BioFocus, and BioMol libraries, approximately 209 were duplicates. Differences in naming, salts of the compounds and chemical nomenclature (e.g., D- *versus* (+)-) in the Excel spreads makes this number an estimate. The Analyticon did not come with a spreadsheet that was exportable to Excel.

The libraries were screened for compounds that inhibited the refolding of thermally denatured luciferase using a high-throughput assay carried out with a slight modification of the method previously described [27,29]. Briefly, compounds were reconstituted in 100% DMSO. Stocks of compounds purchased from Microsource, TimTec, Biofocus and BioMol were reconstituted to a concentration of 1 mg/mL. The stocks were diluted 40-fold into nano-pure water with the compounds being used at a final concentration of 12.5 µg/mL for the assay. Analyticon compounds (0.2 µmol) were reconstituted to a concentration of 4 mM, and were used at a final concentration of 40 µM in the assay. Assays were performed in 96-well microplates. To each well was added 30 µL of the water/DMSO compound solution, 15 µL of the reticulocyte lysate preparation, and 15 µL of the luciferase reagent. The plates were agitated and then allowed to incubate at 25 °C for one to three hours. After the incubation, 60 µL of assay buffer containing luciferin was added to each well. The plates were then read in a Molecular Devices LMaxII^384^ microplate reader, and luminescence was measured in relative light units, with an integration time of 10 s. Compounds that inhibited luciferase refolding by approximately fifty percent or greater were then titrated into a refolding reaction containing native luciferase, to eliminate false positive hits that were direct inhibitors of luciferase as previously described [27,29]. Compounds were classified as a potential Hsp90 inhibitor if they inhibited luciferase refolding by 60% or greater and there was no inhibition of the activity of native luciferase at the compound’s IC_50_ (concentration of the compound that inhibits luciferase refolding by 50%).

To obtain an estimate of a compound’s IC_50_, the compounds were titrated into the assay mixture described above starting at a concentration of 25 µg/mL and a series of three-fold dilutions. The titrations were repeated twice. For a more accurate determination of the IC_50_s for the compounds studied in Section 3.2.1 of this manuscript, a two-fold deletion series of the compounds were used in triplicate, with the experiment being repeated three times.

#### 2.1.5. Statistical Analysis

Z-factors were calculated for the Microsource, TimTec and Analyticon compounds screens as the plates contained an adequate number of positive and negative controls to make the calculation. Robust statistics [32], which minimizes the effects of outliers on the statistical analysis, were used to calculate the median, robust standard deviation (rSD) and the robust percent coefficient of variance (%rCV).

### 2.2. Antiproliferation Assay

MCF7 cells were grown in Gibco Dulbecco’s Modified Essential Medium, supplemented with non-essential amino acids, 2 mM glutamine, and 10% fetal bovine serum. Cells were seeded at 2,000 cells per well in clear 96-well plates containing 100 µL of media per well, and the cells were allowed to attach overnight. The next day, serial dilutions of compounds in DMSO or DMSO control was added to the wells. Cells were then incubated at 37 °C for 48 h. Cell viability [33] was determined using the Promega Cell Titer 96 Aqueous One Solution Cell Proliferation Assay, which makes use of a soluble tetrazolium compound that is converted into a chromophore by living cells. After incubation with compounds, 20 µL of the assay substrate solution were added to the wells, and the plate was incubated at 37 °C for an additional hour. The plate was then read at absorbance at 490 nm using a Molecular Devices Versamax plate reader. Values are expressed as percent DMSO control. All experiments were done in triplicate.

### 2.3. Hsp90-Dependent Maturation of the Heme-Regulated eIF2α Kinase [33]

[^35^S]-Labeled His-tagged HRI was translated by coupled transcription/translation for 20 min in TnT reticulocyte lysate at 30 °C. Drugs or DMSO vehicle control were then added. After an additional 10 min, the reactions (4 µL aliquots) were diluted into 28 µL volumes of heme-supplemented control lysate or heme-deficient lysate, containing the same respective drugs and DMSO vehicle control. The samples were allowed to incubate for another 40 min at 37 °C. The [^35^S]-Labeled His-tagged HRI was then adsorbed from the reactions with NTA-Ni^2+^ agarose resin for 1 h at 4 °C. The agarose resins were the washed four times total, in P50T, P100T 2X, and P50T again. SDS sample buffer was then added to the pellets, the samples boiled, and then separated on an 8% PAGE gel, and transferred to PVDF membrane. Membranes were dried and exposed to X-ray film for autoradiography.

### 2.4. Inhibitor-Dependent Depletion of Hsp90-Dependent Clients from MCF7 Cells

MCF7 cells were grown to confluence in Advanced DMEM/F12 (1:1; Gibco) supplemented with L-glutamine (2 mM), streptomycin (500 µg/mL), and penicillin (100 units/mL) and re-seeded at 0.4 × 10^6^ cells/well/2 mL. Cells were incubated in a humidified atmosphere (37 °C, 5% CO_2_) for 24 h and treated with varying concentrations of compound or 0.5 µM geldanamycin in DMSO (0.25% DMSO final concentration), or vehicle (DMSO) for 24 h. Cells were harvested in cold PBS and lysed using MPER (Thermo Scientific/Pierce, USA) supplemented with protease and phosphatase inhibitors (Roche Applied Science, USA) according to manufacturer’s directions. Lysates were clarified at 14,000 *g* for 15 min at 4 °C. Protein concentrations were determined using the Pierce BCA protein assay kit per the manufacturer’s instructions. Equal amounts of protein (15 μg) were electrophoresed under reducing conditions (10% acrylamide gels), transferred to PVDF, and immunoblotted with the corresponding antibody (Anti-pAkt, -Her2, and -Cdk6 antibodies were from Cell Signaling; anti-actin was from Santa Cruz Biotechnology; and anti-Hsp90 and -Hsp70 were from Enzo Lifesciences). Membranes were incubated with an appropriate horseradish peroxidase-labeled secondary antibody, developed with a chemiluminescent substrate, and visualized.

## 3. Results and Discussion

### 3.1. Natural Product Library Screen

An assay for chaperone-mediated protein renaturation was developed in 1994 using rabbit reticulocyte lysate (RRL) to refold thermally denatured firefly luciferase [34]. RRL had been used for decades for *in vitro* protein synthesis and was known to contain abundant quantities of the heat shock proteins. After the discovery that geldanamycin was an Hsp90 inhibitor, it was also demonstrated to inhibit the refolding of luciferase in RRL, confirming that luciferase renaturation was an Hsp90-dependent process [28]. The assay was demonstrated to be sensitive to compounds that bound and inhibited Hsp70, indicating that the assay could also detect compounds that interacted with other critical components of the Hsp90 chaperone machinery [27]. Subsequently, it was demonstrated that inhibitors that bind to the C-terminal domain of Hsp90 (e.g., novobiocin) also inhibited luciferase refolding in RRL [29]. The assay was then miniaturized and developed as a high-throughput screen for inhibitors of the Hsp90 chaperone machine [29]. Thus, while we generically refer to the compounds discussed below as Hsp90 inhibitors, the molecular target of the potential inhibitor could be any component of the Hsp90 chaperone machinery that is required for the refolding of luciferase.

**Figure 1:**
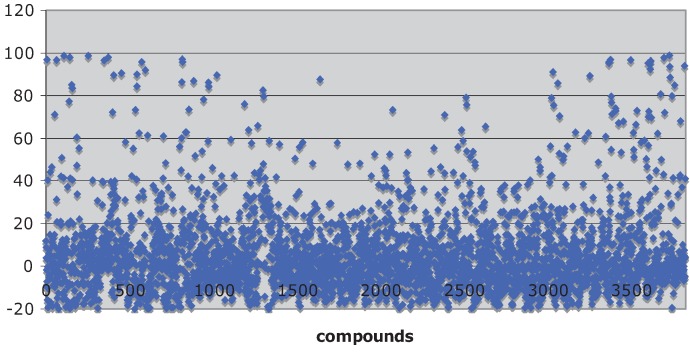
Scatter plot showing the activities of 3,859 of the compounds screened.

Natural product libraries proved to be a fertile ground for identifying inhibitors of the Hsp90 chaperone machine. A scatter plot of percent inhibition of luciferase refolding *versus* 3,859 compounds screened from the TimTec, Biofocus, BioMol and Analyticon libraries is shown in Figure 1 (median = −0.041 ± 13 rSD). The Microsource compounds were part of a previously published screen [29] that had a Z-factor of 0.62 ± 0.09. The Z-factors for the TimTec and Analyticon screens were 0.77 ± 0.18 and 0.64 ± 0.14, respectively. The %rCV for the screens of the TimTec, Biofocus, BioMol and Analyticon screens were 8.9 ± 3.9, 20 ± 6.4, 18 ± 5.0 and 13 ± 6.2, respectively, indicating that the assay had a good signal-to-noise ration.

**Figure 2:**
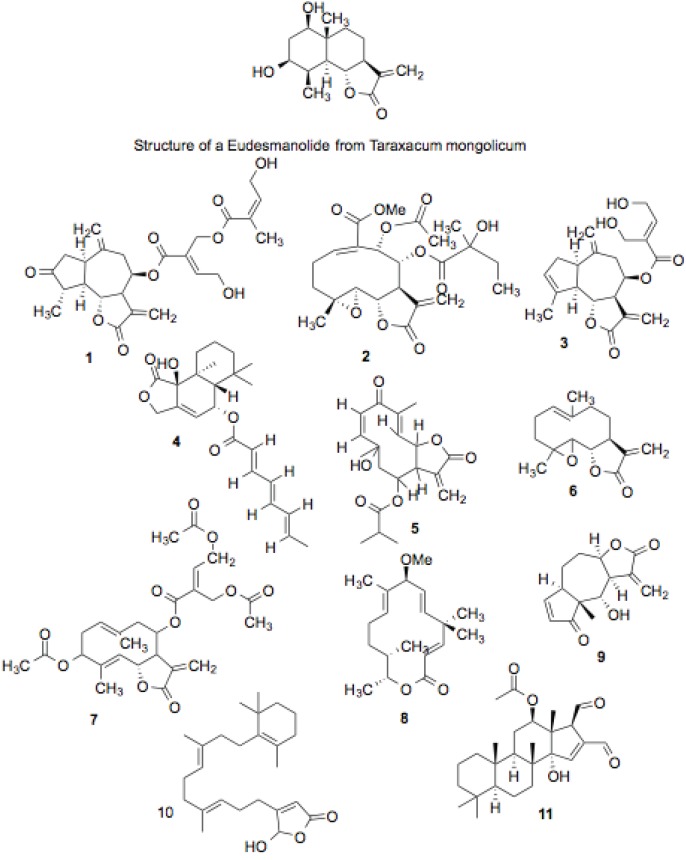
Sesquiterpine lactones, tetracyclic sestorpenoids and sesterpines.

**Figure 3:**
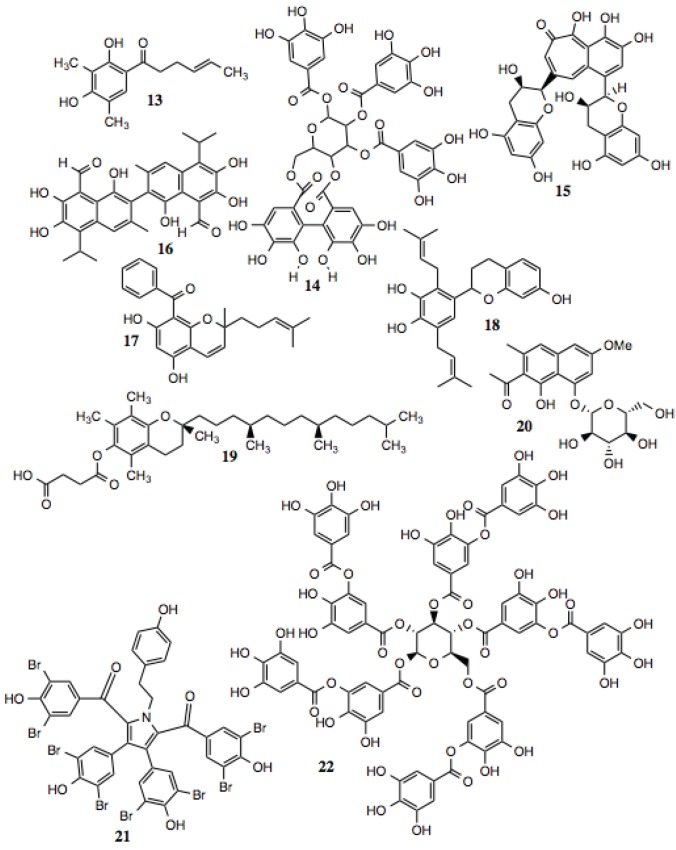
Polyphenols and related compounds.

**Figure 4:**
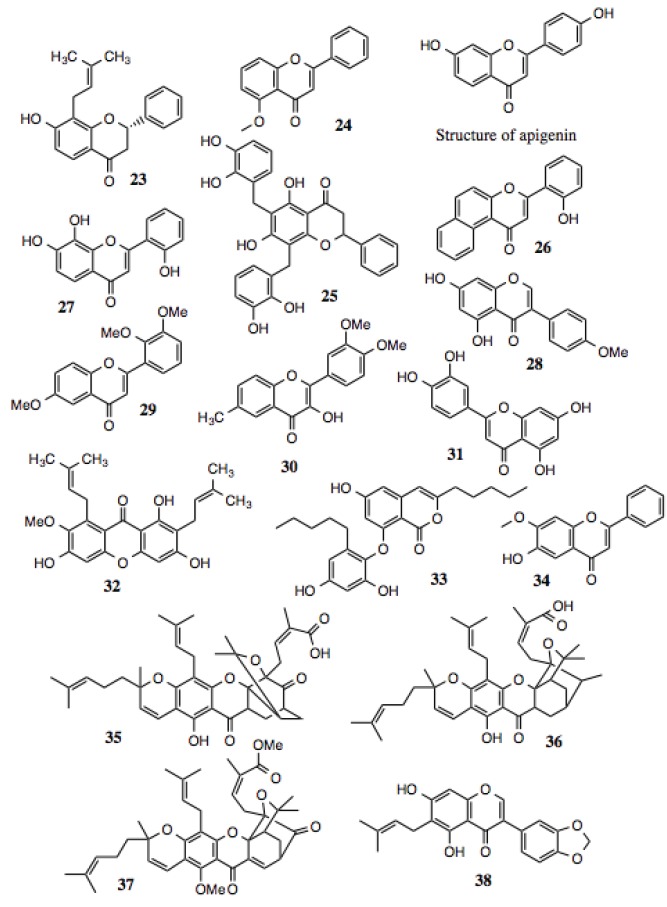
Flavonoids and related compounds.

**Figure 5:**
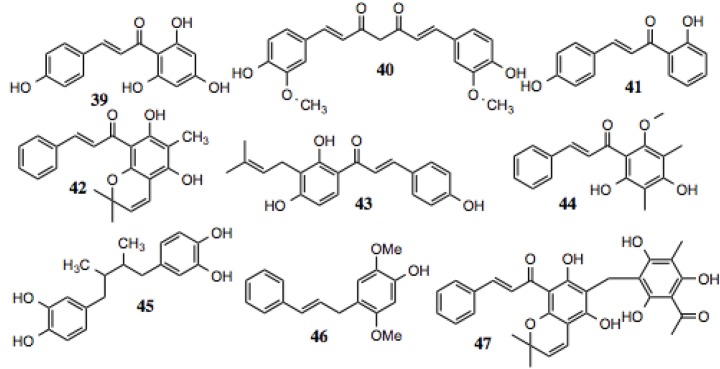
Chalones and related compounds.

The compounds, whose structures are shown in Figure 2, Figure 3, Figure 4, Figure 5, Figure 6, Figure 7, Figure 8, Figure 9, Figure 10 and Figure 11 and are listed in Table 1, Table 2, Table 3, Table 4, Table 5, Table 6, Table 7, Table 8, Table 9, and Table 10, inhibited Hsp90-dependent refolding of luciferase by 60% or greater in the initial screen (a value greater than 4 rSD from the median), without inhibiting luciferase itself, with the exception of the screening of the Microsource compounds which used a cut-off of 70% inhibition [29]. The compounds have been sorted largely by structural classification, although some do not fit well into any particular structural group. Some compounds were present in more than one library. Curcumin (2 screens), gambogic acid (3 screens) and plumbagin (3 screens) were consistently identified as hits. Of the compounds that were present in more than one library, but were not identified as hits, six were present in the Microsource screen that used a cut-off of 70% inhibition, and four compounds failed to be identified as hits because they inhibited luciferase refolding less than the 60% cut-off used for the other libraries. The lone exception was luteolin, which exhibited no inhibitory activity in one of the screens, indicating that its identification as a hit should be viewed with some skepticism.

Because of Hsp90’s role in modulating proteins associated with signal transduction and protein shuttling, its inhibition has widespread and sometimes varied effects within a cell. Depending on the conditions, the physiological manifestations of this inhibition can also vary. Thus, Hsp90 inhibitors display not only anti-proliferative and anti-tumor properties, but they can also display anti-inflammatory, anti-metastatic [20,21,35], immunosuppressant, anti-viral [22], anti-bacterial, anti-fungal [23] and/or anti-malarial [24,25] properties depending on the system investigated. Another property of inhibitors that bind to Hsp90’s N-terminal ATP-binding domain is the induction of a robust heat shock response [10].

The significance of this screen, is that novel compounds not known to have any activity against Hsp90 or its co-chaperones can be implicated to exhibit multiple, seemingly unrelated, medically relevant biological activities. Subsequent to the screen, the literature was mined to identify reports on the physiological effect of potential compounds. As shown below, some of the compounds identified have been specifically shown to inhibit the activities of proteins known to be dependent upon Hsp90 for their function [9,10,20]. These proteins include Akt, STAT-3, Her2 (ErbB2), Insulin-like Growth Factor Receptor (IGFR), Endothelial Growth Factor Receptor (EGFR), telomerase and others. Compounds reported to block the actions of these proteins, or their downstream signaling partners, such as NF-κB, are of special interest. Since Hsp90 is required for the activity of viral polymerases [22,36,37], anti-viral activity is another hallmark manifested by Hsp90 inhibitors [22]. VEGFR1 and 2 [38], and HIF1α [35] are also Hsp90-dependent clients, and as such Hsp90 inhibitors are accordingly anti-angiogenic [39]. Even though they have not been identified as Hsp90 inhibitors, many of the compounds identified from this screen belong to structural families that contain known Hsp90 inhibitors. Below are examples of the biological activities manifested that make each class of compounds, or specific compounds, potential candidates as Hsp90 inhibitors.

#### 3.1.1. Sesquiterpene Lactones, Tetracyclic Sesterpenoids and Sesterpines

Some of the compound hits in this screen belong to the sesquiterpene lactone family of compounds (Figure 2, Table 1). These molecules are characterized by a fifteen-carbon skeleton formed by the union of three isoprene units that contain a lactone group. These compounds are present in many types of plants, and have long been used for various purposes in traditional medicine. Given their effectiveness in the treatment of a wide variety of ailments, and their observed activity on multiple cellular functions and molecular targets, these compounds represent promising candidates as Hsp90 inhibitors.

Sesquiterpene lactones have been grouped into seven general classes according to their structures. They are germacranolides, eudesmanolides, eremophilanolides, guaianolides, pseudoguaianolides, hypocretenolides, and iso-seco-tanapartholides. Compounds with reported biological activity come from all of the groups, although the germacranolides, guaianolides, and pseudoguaianolides appear most prominent [40].

Several sesquiterpene lactones with structures similar to those shown in Figure 1 have demonstrated biological activities. Isodeoxyelephantopin, and its nearly identical analog, deoxyelephantopin, were shown to inhibit the proliferation of mouse fibroblast tumor cells. The two compounds also inhibited DNA replication in both proliferating lymphocytes and tumor ascites [41]. Another pair of compounds fitting into this family, costunolide and eremanthin that have structures similar to compounds **6** and **7**, were extracted from the ornamental plant *Costus speciosus*, and displayed anti-fungal activity similar to the standard anti-fungal, fluconazole, against two species of *Trichophyton*, and somewhat weaker activity against other fungi [42]. As nitric oxide is a mediator of inflammation, a compound’s effect on nitric oxide production is also an indicator of its anti-inflammatory potential, which is especially relevant to this study, as nitric oxide synthase is a well-known Hsp90 client. Eudesmanolides isolated from *Taraxacum mongolicum* inhibit nitric oxide production in RAW 264.7 mouse macrophages [43].

Compound **6**, parthenolide, is from a different class of sesquiterpene lactones, but presents a similar structure. Additionally, it contains a methane group at the same location as the previously mentioned compound, a functional group that is the sole distinguishing feature from another, less active compound in the same study. Compound **4**, an eudesmanolide (MEGxm0_000041), contains a similar moiety at its core, but it also contains an unsaturated eight-carbon fatty acid ester. Two compounds from the plant *Eupatorium lindleyanum*, eupalinolide A and eupalinolide B, are of the germacranolide sub-class. They induced expression of several heat shock proteins, including Hsp70 and Hsp90, in mouse squamous cell carcinoma and melanoma cells. The compounds were also shown to activate HSF1 [44], a potential indicator of Hsp90 inhibition.

Compound **6**, parthenolide, has been identified as an anti-tumor and anti-inflammatory agent, and is currently in clinical trials along with several other sesquiterpene lactones for acute myeloid leukemia, acute lymphoblastic leukemia, and other types of blood and lymph node cancers [40]. Parthenolide’s anti-cancer and anti-inflammatory activities have been attributed to multiple mechanisms. It was shown to inhibit the activation of NF-κB by IKK, even when the kinase was constitutively active [45]. It was also able to sensitize TRAIL-resistant cancer cells by inhibiting STAT3 activation [46].

Compound **9**, helenalin, suppresses NF-κB activation, promotes ROS generation, and induces apoptosis by bypassing Bcl-2 function [47]. Helenalin is cytotoxic against a number of cancer cells, and it also manifests immunosuppressant and anti-inflammatory activity [48]. In addition, helenalin is a potent inhibitor of telomerase [49], which further supports its potential as an Hsp90 inhibitor. Compound **3**, also belongs to this class of compounds, and presents a similarity in the attached moieties.

Grouped with these compounds are two additional hits. Luffariellolide, a sesterterpene from a marine sponge, is cytotoxic to breast cancer cells, and inhibits the activation of the Hsp90-dependent protein HIF-1α [50]. Compound **11**, 12-epi-scalardial, a tetracyclic sesterpenoid, inhibits EGFR-mediated activation of Akt [51].

**Table 1:** Sesquiterpine lactones, tetracyclic sesterpenoids and sesterterpenes.

#	Location and/or name	IC_50_ (µM)	Properties
1	Guaianolide	~60	
2	Germacranolide	~40	
3	Guaianolide	~40	
4	Eudesmanolide: MEGxm0_000041	~60	Cytotoxic to L5178Y lymphoblastic, PC12 neuroendrocrine, HeLa cervical cancer cells [52]
5	Tagitinin C	~10	Anti-malaria [53]
6	Parthenolide	>100	Anti-tumor, anti-inflammatory [40,45,46]
7	17-C4	~30	
8	16-H2	~10	
9	Helenalin	~80	Inhibition of NF-κB and suppression of Bcl-2-mediated resistance to apoptosis [47]; anti-leukemic [54]; inhibition of telomerase [49]; induction of autophagy and cell cycle arrest [55]
10	Luffariellolide	~10–20	Cytotoxic, inhibits HIF-1α [50]
11	Scalaradial, 12-epi-	~40–60	Inhibits EGFR activation of Akt independent of PLA2 [51]

#### 3.1.2. Polyphenols

Polyphenols are defined as compounds that contain multiple phenolic moieties and often are poly-hydroxylated. This family of compounds is large, and contains multiple subtypes. Polyphenols generally exhibit anti-oxidant activity and can protect against ROS *in vitro*. The actual mechanisms behind these activities, however, have not been fully evaluated. Several of the compounds shown in Figure 3 and listed in Table 2 inhibit Hsp90-dependent proteins, and manifest cytotoxic, anti-proliferative, anti-inflammatory, and anti-viral activities, among others, that might be explained by their ability to inhibit Hsp90. Theaflavin (compound **15**) has recently been reported to be an inhibitor of Hsp90 [56].

**Table 2:** Polyphenols and related compounds.

#	Location and/or name	IC_50_ (µM)	Properties
13	2'3'-dihydrosorbicillin	>100	Moderate cytotoxic activity against cancer cell lines [57].
14	Tellimagrandin II	~70	Anti-HIV [58]; suppression of sarcoma tumor cell growth [57].
15	Theaflavin	~25	Anti-viral, anti-inflammatory [59]; anti-proliferative against leukemia cells via Akt down-regulation via Hsp90 inhibition [56]; inhibits NF-κB and MAPK signaling [60];
16	Gossypol	~50	Anti-oxidant; broad anti-cancer activity; anti-viral; anti-protozoan; anti-bacterial; contraceptive [61].
17	Similar to catechin (CID 10670714)	~50	
18	Flavan-3-ol (AC1MR5D9, CID 3512639)	~60	
19	(+)-α-Tocopherol acid succinate	~50	
20	Torachrysone 8-O-Glucoside (CID 11972479)	~25	Anti-oxidant [62]; inhibits LPS-induced NO production and NF-κB activation [63].
21	Polycitone A	~10–20	
22	Tannic acid	~5	

#### 3.1.3. Flavonoids

Flavonoids (Table 3, Figure 4) represent a large and diverse group of compounds that reside within the class of polyphenols. Flavonoids comprise approximately one half of all identified polyphenolic compounds. Aside from their aromaticity, the molecules have no unifying characteristic, except that they contain two or more six-membered rings, as well as at least one oxygen atom in the form of an ether or ketone. Many of these compounds contain multiple ketones and hydroxyl groups. This family of compounds is abundant in a number of substances used in traditional medicine. These substances have been shown to exhibit activities against allergy, inflammation, infection, tumors, diarrhea, and others. They have also been credited with wound healing and other beneficial properties. As ubiquitous as flavonoids are in plants, they are found in many foods. Examples are quercitin, epigallocatechin gallate (EGCG), resveratrol, and others.

Several hits from the screening belong to the flavonoid family (Table 3, Figure 4). Some of the compounds contain the typical bicyclic core along with a benzene ring fused to a pyran or pyrone, as well as a phenyl group attached to the flavan, isoflavan, or neoflavan. They also contain phenyl or aliphatic groups of varying saturation and oxygen incorporation. Additionally, some of the compounds fall into the subgroup of flavonoids known as chalcones (Table 4, Figure 5), which are metabolic precursors to flavonoids. These chalcones are characterized by two benzenes bridged by a 2-propen-1-one group.

Flavonoids are found throughout the plant kingdom. They have been used for the treatment of disease for centuries. Many flavonoids also demonstrate anti-microbial activity. Argentinian folk medicine has made use of a plant containing the glycosylated flavonol, quercetagetin-7-arabinosyl-galactoside, for the treatment of infectious diseases [64]. In another study, epigallocatechin gallate, a type of flavonoid found abundantly in green tea, demonstrated strong anti-bacterial activity, resulting from damage to the lipid bilayer [65]. However, EGCG has also been shown to bind Hsp90 and to induce degradation of Hsp90-dependent substrates [66].

**Table 3:** Flavonoids.

#	Location and/or name	IC_50_ (µM)	Properties
23	Glabranine (CAS 41983-91-9)	>75	Anti-viral [67].
24	5-Methoxyflavone	~100	
25	MolPort-005-945-561 (CID 4560115)	~70	
26	2'-hydroxy-b-naphtho-flavone	~350	
27	7,8-dihydroxy-2-(2-hydroxyphenyl)chromen-4-one	~10	
28	Biochanin A (5,7-dihydroxy-3-(4-methoxyphenyl)-chromen-4-one)	>90	Anti-proliferative, anti-inflammatory, cytotoxic; inhibits iNOS expression, MAPK phosphorylation and NF-κB activation [68].
29	2',3',6-Trimethoxyflavone (2-(2,3-Dimethoxyphenyl)-6-methoxy-4H-chromen-4-one)	>90	
30	3',4'-Dimethoxy-3-hydroxy-6-methylflavone	~30–80	
31	Luteolin	>100	Induction of unfolded protein response and apoptosis in neuroblastoma [69]; inhibits LPS-activated, Akt-mediated activation of NF-κB in macrophages [70]. Anti-tumor activity through EGFR pathway suppression in breast cancer cells [71]. Shown to inhibit Hsp90 [72].
32	Mangostin [1,7-bis(3-methylbut-2-enyl)-3,6,8-trihydroxy-2-methoxy-xanthen-9-one]	~60	Xanthanoid–Induces cell cycle arrest and apoptosis in colon [73] and prostate cancer cells [74]. Blocks activation of MAPK and Akt pathways [75].
33	MolPort-001-742-269 (CID 38356110)	~65	
34	6-Hydroxy-7-methoxyflavone	~45	
35	Gambogic acid	~2	Demonstrated to inhibit Hsp90 [33,76].
36	Tetrahydrogambogic acid	~10	
37	Dimethyl Gambogate	~2	
38	Derrubone	~0.2	Inhibitor of Hsp90 [77].

Over the course of twenty years, apigenin, a simple flavone, was assayed for its anti-bacterial activity, and was found active against more than fifteen types of pathogenic bacteria, including *S. aureus*, MRSA, *E. coli*, *P. aeruginosa*, and *K. pneumonia* [78]. In addition to anti-microbial activity, these compounds also demonstrate anti-cancer activity. For example, apigenin (Figure 3) exhibited strong *in vitro* anti-tumor and anti-angiogenic activity against human lung, prostate, and ovarian cancer cells. In each of these cases, the expression of VEGF and HIF-1alpha were suppressed [79,80], both of which are Hsp90-dependent clients.

Among the hits from this screen, compounds **23**, **24**, **27**, **29**, **30** and **34**, contain the same flavone core as apigenin. Two contain a flavonone core that is nearly identical, except for saturation of the C-2 double bond found in flavones. The physiological effects of several of the flavonoid hits implicating them as possible Hsp90 inhibitors are noted in Table 3.

#### 3.1.4.Chalcones

Chalcones are a structurally distinct subclass of flavonoids from which several hits were identified (Table 4, Figure 5). Chalcones share many of biochemical characteristics with other flavonoids, as they exhibit anti-fungal [81], anti-inflammatory [82], anti-tumorogenic [83], anti-HIV, and anti-plasmodial activities [84], amongst others (Table 4). A number of cellular proteins were identified as targets for the chalcones, many of which are known to be dependent upon Hsp90 (Table 4), including Akt, NF-κB, mTOR, STAT3, HIF-1α, iNOS, and others [85].

#### 3.1.5. Pentacyclic Triterpenoids

Three pentacyclic triterpenoids were identified in the screening (Table 5, Figure 6), celastrol, its methyl ester and anthothecol. Celastrol is a well-established Hsp90 inhibitor [86]. Anthothecol is a limonoid related to degunin. Degunin has also been identified as an inhibitor of the Hsp90 machine [87,88].

**Table 4:** Chalone compounds.

#	Location and/or name	IC_50_ (µM)	Properties
39	Phloretin	~35–90	Induction of apoptosis in breast cancer cells via Bcl-xL degradation [89].
40	Curcumin	~70	Anti-proliferative, anti-tumor, anti-inflammatory via suppression of NF-κB activation [90]; reported Hsp90 inhibitor [91].
41	2',4-Dihydroxychalone	~120	
42	[6-methyl-5, 7-dihydroxy-2,2-dimethylchromen-8-yl]-3-phenylprop-2-en-1-one Similar to rotterlin and catechin	~60	
43	(CID 193568) Corylifolinin; isobacachalone	~50	Inhibition of LPS-induced NO production [92].
44	Dimethyl cardamonin (CID 10424762)	~70	Antibacterial; anti-fungal [93,94]; anti-proliferative via cell cycle arrest; anti-inflammatory via blocking NF-κB activation [95,96,97,98].
45	Nordihydroguaiaretic acid	~35–80	Phase II study for effect on prostate cancer. Increased doubling time of PSA. Thought to inhibit IGF1R and HER2 [99]. Repressed breast tumor growth via mTORC1 inhibition [100].
46	Violastyrene	~20	
47	Rottlerin	~60	Anti-proliferative [101,102]; cytotoxic to pancreatic cancer cells via PI3K/Akt/mTOR inhibition [103]; inhibits NF-κB; STAT and amyloid aggregation [104].

**Table 5:** Pentacyclic triterpenoids.

#	Location and/or name	IC_50_ (µM)	Properties
48	Celastrol	~2	Hsp90 inhibitor [86].
49	Celastrol methyl ester	~2	
50	Anthothecol	~6	Antimalarial [105].

**Figure 6:**
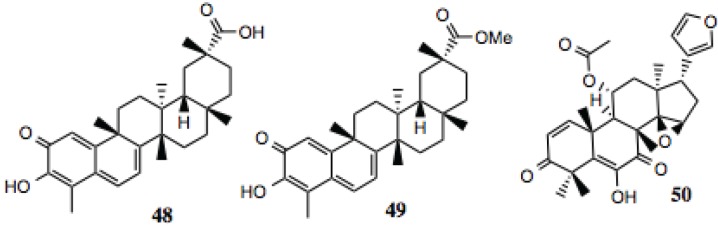
Pentacyclic triterpenoids.

#### 3.1.6. Alkaloids

**Figure 7:**
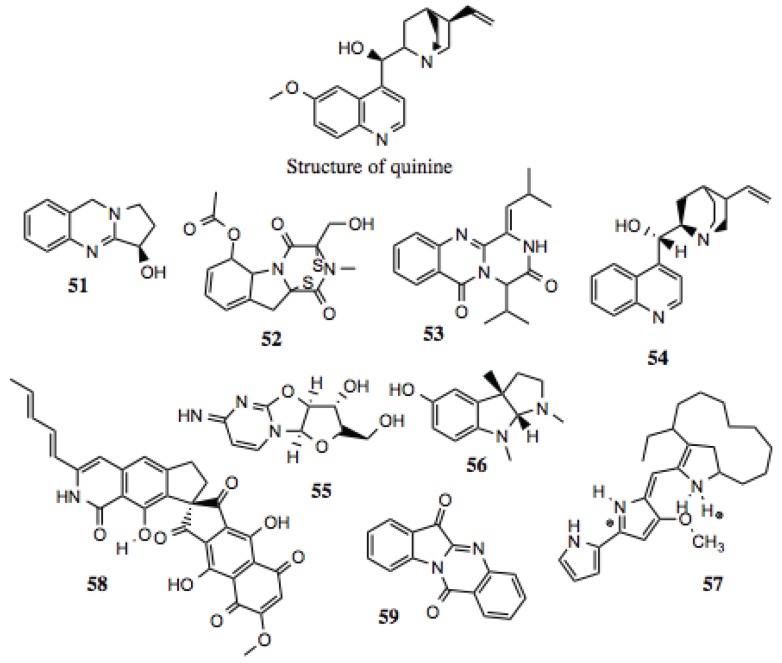
Alkaloid compounds.

Alkaloids are broadly defined as containing basic nitrogen atoms within their ring structures. As this definition includes a large number of potential compounds, the family is further broken down into smaller subdivisions. Regardless, the compounds within the family that have demonstrated biological activities are diverse, with no single structure or group of structures requisite for activity. For the purposes of this screen, alkaloids were regarded as molecules that contain a cyclic nitrogen atom. Similar to other groups identified in this screen, alkaloids (Table 6, Figure 7) demonstrate a wide range of medically relevant bioactivities, which include anti-tumor, anti-hypertensive, anti-depressant, anti-microbial, anti-inflammatory, and other activities [106], as well as inhibiting the function of some well known Hsp90-dependent proteins (Table 6). A well-known example of a medicinal alkaloid is quinine (Figure 7), isolated from the tropical medicinal plant *Cinchona succirubra*, which has been used to treat malaria for hundreds of years [53].

#### 3.1.7. Benzylisoquinoline Alkaloids

Benzylisoquinoline alkaloids (Table 7, Figure 8) comprise a subset of alkaloid compounds characterized by an aromatic, tetracyclic skeleton, containing three benzene moieties, and a fourth cycle containing the alkaloid nitrogen. Most of the compounds shown in Figure 7 are aporphines that are highly similar, and differ only by the presence or location of a hydroxyl, methoxy, or keto group. Some, however, incorporate an additional ring, or exist as a dimer of two aporphine molecules. While there is little known about most of the aporphine compounds identified in this screen, some have no prior designation. One example is glaucine, which manifests a host of activities *in vitro*, including relaxation of bronchia via inhibition of its contraction, reduction in superoxide generation in stimulated polymorphonuclear leukocytes and eosinophils, reduction of elastase release, leukotriene production, and intracellular Ca^2+^ in PMN’s, platelet aggregation, and eosinophil peroxidase release. These effects make glaucine a likely candidate for the treatment of bronchiodilation and inflammation [107].

**Table 6:** Alkaloid compounds.

#	Location and/or name	IC_50_ (µM)	Properties
51	Peganine (CID 72610)	~130	Modest anti-proliferative and cytotoxic activity [108]; anti-mycobacterial [109]; anti-leishmania [110,111].
52	Gliotoxin acetate (CID 21127802)	~15	Anti-viral; anti-mycobacterial; inhibition of NF-κB; anti-leukemic; anti-tumor [112,113,114].
53	19-A7: (*Z*)-4-isopropyl-1-(2-methylpropylidene)-1,2-dihydro-6*H*-pyrazino[2,1-*b*]quinazoline-3,6(4*H*)-dione	~60	
54	(+)-Cinchonine	~30	Circumvention of P-glycoprotein mediated multi-drug resistance [115].
55	(2*S*,3*R*,3a*S*,9a*R*)-2-(hydroxymethyl)-6-imino-2,3,3a,9a-tetrahydro-6*H*-furo[2',3':4,5]oxazolo[3,2-*a*] pyrimidin-3-ol	~50	
56	(−)-Eseroline	~50–90	
57	Metacycloprodigiosin; Streptorubin	~10	Cytoxic activity against cancer cell lines [116,117].
58	Fredericamycin	~3	Cytotoxic, anti-bacterial, anti-fungal, anti-tumor activities; topoisomerase inhibitor; cell cycle arrest [118,119].
59	Tryptanthrin	~15	Induced apoptosis in human leukemia cells; suppression of NO production [120].

Aporphines also demonstrate *in vitro* anti-viral activity. A number of compounds inhibit polio-virus infection of cultured mammalian cells by 50% at low micromolar concentrations [121,122]. Additionally, the aporphines dicentrine, glaucine, corydine, and apomorphine, which are analogs of the aporphines presented in this study, demonstrate anti-proliferative activity against mouse leukemia, melanoma, bladder cancer, and colon cancer cells [123].

As discussed earlier, Hsp90 contains a distinct ATP-binding domain, the specificity of which has been exploited for the development of inhibitors. This domain contains a Bergerat fold, and is shared by only a few protein families, which include DNA gyrase, a type II topoisomerase. Some aporphines are inhibitors of topoisomerase II, increasing the likelihood that highly similar compounds from this screen are also Hsp90 inhibitors. One such example is liriodenine (Figure 7), which manifests activity against human cancer cells [124], Gram-positive bacteria, yeast, and fungi [125,126,127].

**Table 7:** Benzylisoquinoline Alkaloids.

#	Location and/or name	IC_50_ (µM)	Properties
60	Sanguinarine	~70	Induces cell cycle arrest and apoptosis in human cervical cancer cells [128]; anti-inflammatory [129]; anti-fungal [130].
61	3-hydroxymethyl-glaucine	~80	
62	Thaliporphine; thalicmidine	~9	Inhibits the activity of LPS-induced NOS; inhibits LPS-induced nuclear translocation of NF-κB [131].
63	Isoboldine	~30–80	Anti-viral [121].
64	Bracteoline	~80	
65	7-oxoglaucine	~80	Anti-plasmodial [132]; anti-inflammatory [133].
66	7-Formyl-dehydroglaucine	~1–3	
67	Dehydroglaucine	0.9	Anti-microbial, some anti-fungal activity [126].
68	Dehydroglaucine dimer	0.1–0.4	
69	Glaucine derivative	>30	
70	Dehydroglaucinylphenone	~7–20	
71	Berberine	~40	Anti-tumor, anti-metastatic, inhibits HIF1α [129,134,135,136].
72	3-Formyl-glaucine	~8–20	
73	Dihydrosanguinarine derivative	~40	Anti-fungal [130].

**Figure 8:**
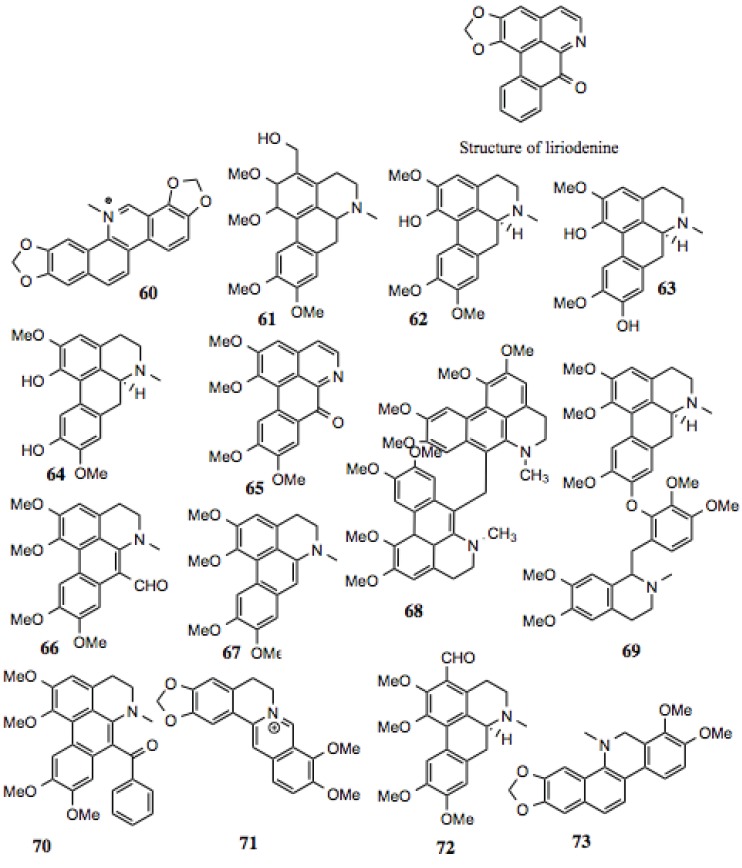
Benzylisoquinoline Alkaloids.

#### 3.1.8. Cyclic Peptides

The McAlpine Laboratory has investigated the anti-cancer and Hsp90-inhibiting activities of naturally occurring macrocyclic peptides. These molecules and their derivatives have demonstrated modest activity against a number of cancers [137]. The rationale behind the pursuit of these particular scaffolds is that similar compounds have been identified as antibiotics and anti-fungals, while maintaining anti-cancer activity. The polypeptide nature of these compounds may confer the ability to mimic hydrophobic portions of client proteins. Four of the compounds identified from this screen (Table 8, Figure 9) contain moieties that could mimic hydrophobic amino acids. Of particular interest is antibiotic A83586 C (compound **75**), which manifest potent anti-proliferative activity against cancer cells [138].

**Table 8:** Cyclic peptides.

#	Location and/or name	IC_50_ (µM)	Properties
74	Tyrothricin	~5	Antibiotic [139].
75	Antibiotic A83586C	~10	Anti-proliferative activity against cancer cells [138].
76	P12	~10	
77	Cyclopeptide L-156373	~10	

**Figure 9:**
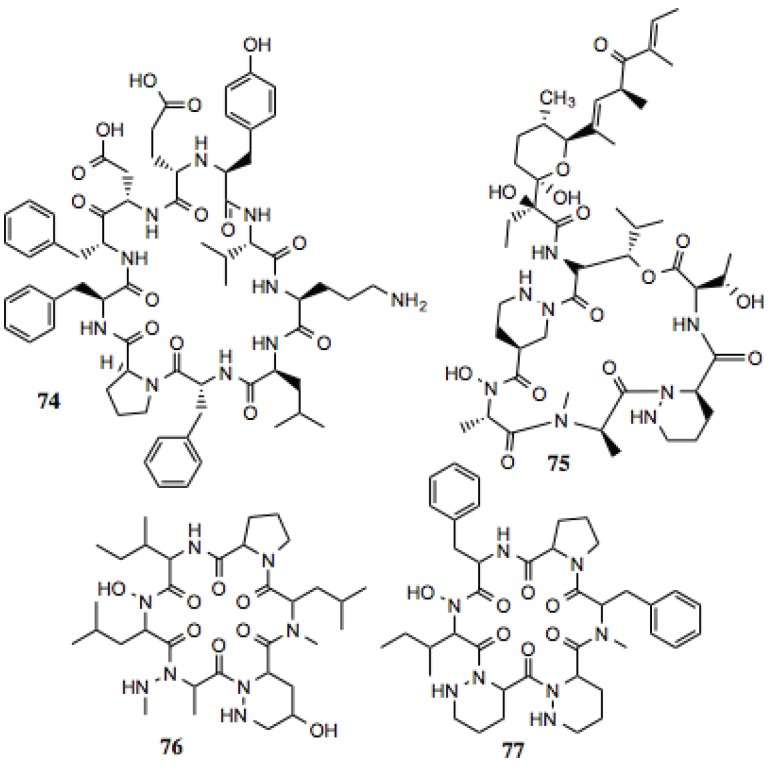
Cyclic peptides.

#### 3.1.9. Quinones

Quinones represent an extremely large and diverse family of compounds. Essentially, the only thing that differentiates a quinone from other classes of compounds is the presence of two keto groups on an unsaturated six-membered ring. As such, an enormous array of functional and structural groups that decorate this motif is possible. Generally, quinones are redox-active, making them promising compounds with which to treat cancer, but also potential liabilities that may arise as a consequence of this activity. The production of reactive oxygen species resulting from exposure to quinone-containing compounds is a process that is potentially destructive to any cell. It has not been established whether the redox potential of these compounds contributes to the anti-cancer activity by increasing their ability to inhibit Hsp90, or by increasing cellular stress alongside Hsp90 inhibition. It should be noted, that reduction of 17-DMAG and 17-AAG to the corresponding hydroquinones resulted in increased Hsp90 inhibitory activity. [140]

**Figure 10:**
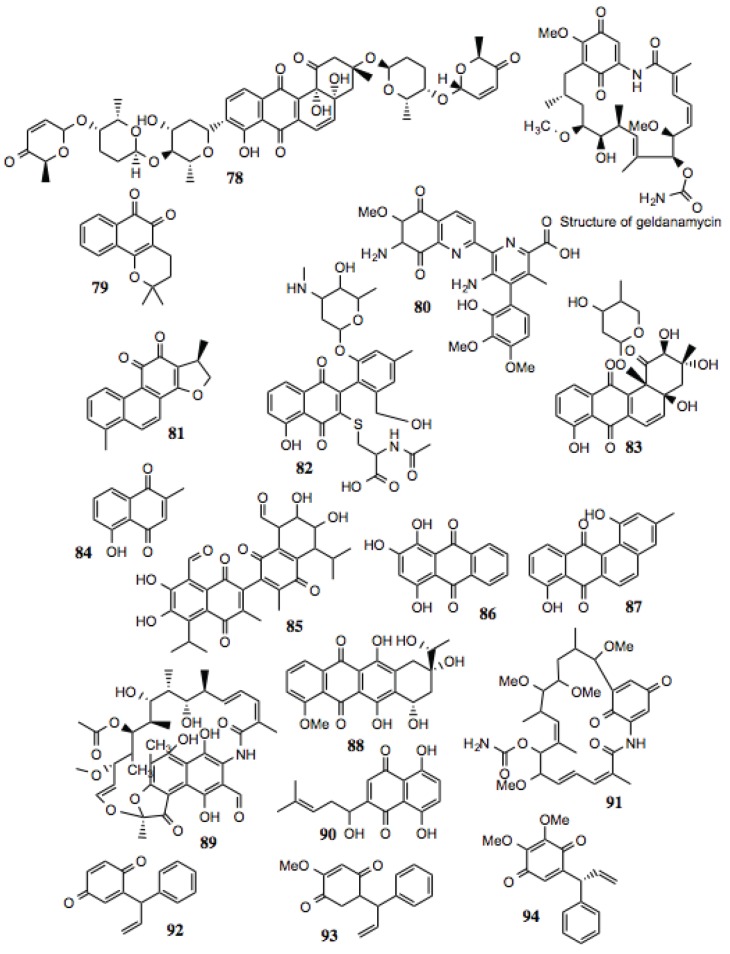
Quinones.

Some well-established Hsp90 inhibitors, such as geldanamycin (Figure 10) and its derivatives, contain quinone moieties. Consequently, we have observed reticulocyte lysate treated with these inhibitors to possess a distinctive dark red color, attributable to met-hemoglobin formation resulting from the oxidative activity of such compounds. Similarly, geldanamycin has been shown to generate reactive oxygen species *in vitro* and in cell culture [141]. Structural studies demonstrate direct binding of these compounds to the ATP-binding site at the N-terminus of Hsp90. Not surprisingly, the antibiotic, rifamycin, contains a reduced quinone moiety within a similar anthroquinone ansamycin structure, and has similar activity as geldanamycin. Seventeen quinones were identified in this screen (Table 9, Figure 10). As indicated in Table 9, a number of these compounds display anti-cancer, anti-trypanosomal, anti-viral, and anti-inflammatory activities, as well as having the capacity to inhibit the activities of several well-known Hsp90-dependent proteins.

#### 3.1.10. Other Compounds

**Figure 11:**
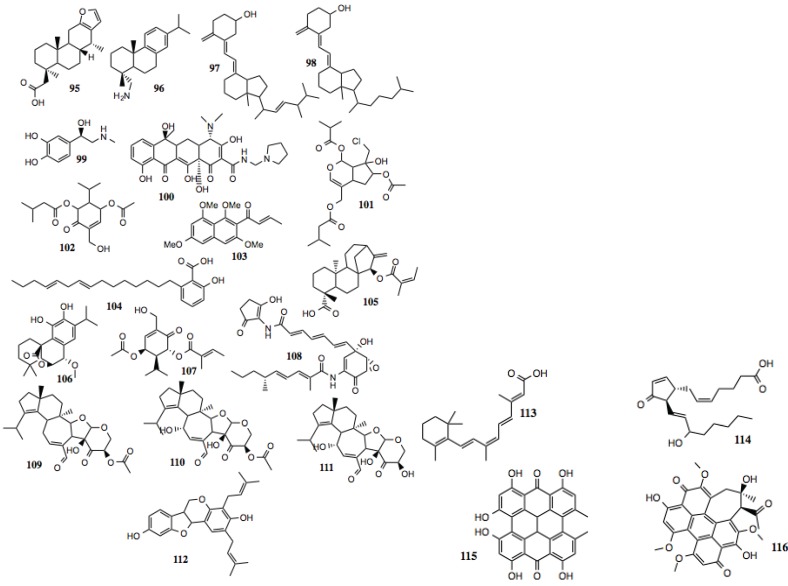
Other miscellaneous compounds.

Compounds identified in this section (Figure 11, Table 10) do not fit well into any of the previously described families. Some of these are known biochemicals, such as Vitamin D2, 9-cis-retionioc acid, prostaglandin J2 and L-adrenaline, and have not been implicated as Hsp90 inhibitors, although Vitamin D2 [142,143] and retinoic acid [144,145] have demonstrated anti-cancer activity. Others are more exotic and little, if anything is known about their mechanism of action. Recently, the anti-cancer activity of hypericin has been tied to its ability to inhibit Hsp90 stabilization of HIF-1α [146].

**Table 9:** Quinones.

#	Location and/or name	IC_50_ (µM)	Properties
78	Vineomycin A1	~100	
79	β-Lapachone (CID 3885)	~20	Anti-cancer; anti-trypanosomal activity; anti-viral [147,148,149,150,151,152,153,154,155].
80	Streptonigrin (CID 5351165)	~15	Disrupts NF-κB activation; antibacterial, antifungal, antiviral, anti-glioma [156,157,158,159,160,161].
81	15.16-Dihydrotanshinone	~80	Anti-cancer; inhibition of HIF-1α; depletion of Bcl-2 [162,163,164].
82	2-A5: 1,4-Napthoquinone derivative	~10	
83	30-D10: 9,10-Anthraquinone derivative	~2	
84	Plumbagin	<0.05	Widely studied anti-cancer activity; reported to target EGFR, STAT-3, Akt, and NF-κB pathways [165,166].
85	7-[8-formyl-6,7-dihydroxy-3-methyl-5-(methylethyl)-1,4-dioxo(2-naphthyl)]-2,3- dihydroxy-6-methyl-4-(methylethyl)-5,8-dioxonaphthalenecarbaldehyde	~6	
86	1,2,4-trihydroxyanthracene-9,10-dione	~40–100	
87	Tetrangulol G2	~20	
88	Dihydodaunomycinone; Leukaemomycin-D	>60	
89	3-Formyl Rifamycin SV	~15–35	Inhibition of Vaccinia virus assembly [167].
90	Shikonin	~75	Anti-inflammatory, anti-tumor, and wide-ranging activities reported [166,168].
91	Herbimycin	~40	Hsp90 inhibitor [1,169,170,171].
92	Dalbergione	~2	
93	4'-Methoxydalbergione	~5	Anti-trypanosomal [172].
94	3'4'-Dimethoxydalbergione	~5	Anti-trypanosomal [172].

**Table 10:** Other compounds.

#	Location and/or name	IC_50_ (µM)	Properties
95	2-((4*R*,6a*S*,7*R*,11b*R*)-4,7,11b-trimethyl-1,2,3,4,4a,5,6,6a,7,11,11a,11b-dodecahydro-phenanthro[3,2-*b*]furan-4-yl) acetic acid	>75	
96	(+)-Dehydroabietylamine; Leelamine	~30–75	
97	Vitamin D2	~60	
98	Vitamin D2 metabolite	~60	
99	L-Adrenaline	~55	
100	Rolitetracycline	~20	
101	MolPort-005-945-572	~50	
102	CID 45359640	~50	
103	(*E*)-1-(1,3,6,8-tetramethoxy- naphthalen-2-yl)but-2-en-1-one	~5	
104	CID 53984538: 8,11- anacardic acid	~10	Anti-cancer; induction of the UPR [173,174].
105	(4*R*,7*R*,11b*S*)-4,11b-dimethyl-7-(((*Z*)-2-methylbut-2-enoyl)oxy)-8-methylenetetradecahydro-6a,9-methanocyclohepta[*a*]naphthalene-4-carboxylic acid	~40	
106	(4b*R*,9*S*,10*S*)-3,4-dihydroxy-2-isopropyl-10-methoxy-8,8-dimethyl-6,7,8,8a,9,10-hexahydro-5*H*-9,4b-(epoxymethano) phenanthren-12-one	~30	
107	CID 13818684	~15	
108	Manumycin A	~30–50	Inhibition of STAT-3 and telomerase; down-regulation of Akt and MEK; anti-cancer [175]; induces autophagy [176].
109	Striatal A	~2	
110	Striatal B	~2	Inhibition of growth of multiple cancer cell lines- NCI cell line growth inhibition assay [PubChem CID 329431].
111	Striatal C	~2	
112	CID 45360154	~50	
113	9-*Cis*-retinoic acid	~30–50	
114	Prostaglandin J2	~75–100	
115	Hypericin	~50–75	Inhibits HIF-1α [146].
116	Hypocrellin A	~5–10	

### 3.2. Further Characterization of Select Putative Inhibitors

The HTS of commercially available natural product libraries identified over a 100 compounds as potential Hsp90 Inhibitors. This raises the question inherent to hits identified in HTS: which of the compounds should one select for further characterization using secondary screens? Of these compounds, derrubone [77], compounds containing the 1,4-naphthoquinone scaffold [177], and gambogic acid [33] have been characterized, in addition to silybin [178], which was identified as an Hsp90 inhibitor by mining the literature. Based on the results from our high-throughput screen and data mining, anthothecol and rottlerin were selected for further characterization. In addition, during the search through the literature for natural products that displayed broad biological activities, garcinol, a gambogic acid-like compound, and piplartine/piperlongumine, a polyphenol chalcone-like compound were identified as potential Hsp90 inhibitors. An additional consideration in the selection of rottlerin, garcinol, and piplartine/piperlongumine was that the literature indicated that these compounds were being discussed as possible candidates for clinical trials.

**Figure 12:**
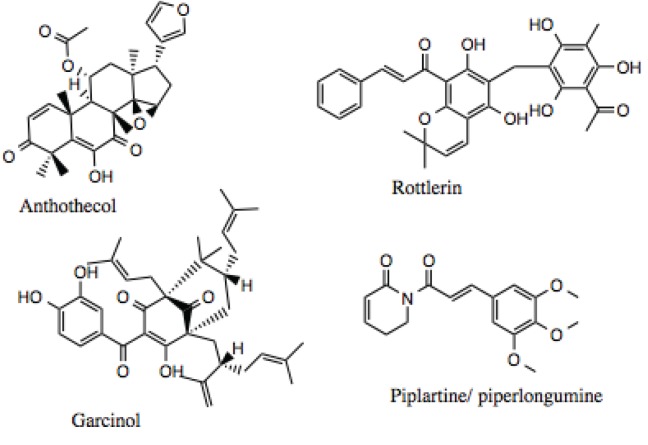
Structure of anthothecol, rottlerin garcinol and piplartine/piperlongumine.

Of the four compounds chosen for additional investigation, rottlerin (Figure 5 and Figure 12) is the best studied, and, as noted above has been discussed as a possible candidate for clinical trials [101]. It has been used in traditional medicine and demonstrates many physiologically significant biological activities. Rottlerin is isolated from the tropical tree *Mallotus philippinensis* and displays cytotoxicity against a number of cancer types, including lung, breast, lymphocytic leukemia, and multiple myelomas. While the activity of rottlerin was initially attributed to inhibition of PKCδ [101], this mechanism of action has largely been called into question [101,103]. Rottlerin appears to inhibit a combination of signal transduction pathways at multiple levels [101], making it a good Hsp90 inhibitor candidate. For example, an array of human malignant tumor cells was treated with rottlerin, and all lines were found to undergo apoptosis mediated by Death Receptor 5 (DR5) [179]. Rottlerin has also been reported to inhibit the kinases PRAK, MAPKAP-K2, Akt, and CaMK [180].

Anthothecol (Figure 6 and Figure 12) is a limonoid natural product isolated from the *Khaya anthotheca* tree, and possesses low micromolar activity against the growth of *Plasmodium falciparum* in erythrocytes [105], which is another property manifested by Hsp90 inhibitors. The compound’s structure is similar to that of the Hsp90 inhibitor, gedunin [88], but it has approximately 10-fold higher activity, as will be demonstrated below.

Garcinol (Figure 12) was chosen for further study, because it demonstrated the ability to induce apoptosis in a number of cancers, including breast, colon, kidney, prostate, leukemia [181], pancreatic [182], and others. The anti-cancer activity of garcinol has been attributed, in part, to its inhibition of STAT and NF-κB signaling [183,184]. Additionally, garcinol can inhibit angiogenesis through down-regulation of Prostaglandin E2, VEGF, and IL-8 [182], which are bonafide Hsp90-dependent clients. Garcinol also exhibits anti-oxidant and anti-inflammatory properties, as it inhibits the production of ROS and nitric oxide [185].

Piplartine (a.k.a., piperlongumine, Figure 12) has been shown to suppress platelet-derived growth factor (PDGF) signaling [186], and inhibit the proliferation of prostate cancer cells through depletion of the androgen receptor, a well-known Hsp90 client protein [187]. A recent review noted that the pharmacological activities for piplartine reported in the literature include cytotoxic, anti-tumor, anti-angiogenic, anti-metastatic, anti-bacterial, anti-fungal, anti-leishmanial, anti-trypanosomal, and anti-schistosomal activities among others [188].

In our study, we provide evidence that further implicates these compounds as inhibitors of the Hsp90 complex. We show that, in addition to inhibiting the proliferation of cancer cells, these compounds also inhibit the Hsp90-dependent folding of thermally denatured luciferase in a dose-dependent manner, block the Hsp90-dependent maturation of the heme-regulated eIF2α kinase (HRI), and deplete cells of Hsp90-dependent clients.

#### 3.2.1. Inhibition of Hsp90-Mediated Refolding of Denatured Luciferase

Anthothecol and rottlerin were identified as potential Hsp90 inhibitors by their ability to inhibit refolding of thermally denatured luciferase in screens of natural compound libraries. To more accurately define their inhibitory activity, the compounds were titrated into the assay in a three-fold dilution series. The compounds inhibited refolding of luciferase in a concentration-dependent manner with low µM IC50s (Table 11). Similarly, garcinol, and piplartine display the ability to inhibit the refolding of luciferase at micromolar concentrations in reticulocyte lysate (Table 11), implicating them as Hsp90 inhibitors.

**Table 11:** IC50 values for inhibition of luciferase refolding and proliferation of MCF7 cells.

Compound	IC_50_ luciferase refolding (µM) ^1^	IC_50_ MCF7 proliferation (µM) ^2^
Anthothecol	12 ± 2	0.5 ± 0.06
Rottlerin	63 ± 5	7 ± 3
Garcinol	12 ± 5	4 ± 0.6
Piplartine	80 ± 7	10 ± 3

^1^ Anthothecol, rottlerin, garcinol and piplartine were titrated into wells containing denatured luciferase and reticulocyte lysate. After a two-hour incubation period, assay buffer containing luciferin was added, and relative luminescence was measured. IC_50_ is the concentration of the compound that inhibited luciferase refolding by 50% compared to the DMSO control; ^2^ MCF-7 cells were treated in culture with anthothecol, rottlerin, garcinol and piplartine and DMSO as a control. Proliferation was assessed at 48 h using an MTS assay. Proliferation is defined as the colorimetric intensity difference between wells treated with DMSO and wells treated with the compounds.

#### 3.2.2. Compounds Inhibit Proliferation of Human Cancer Cells

While piplartine, rottlerin and garcinol have well characterized anti-proliferative properties, there were no reports in the literature with regards to the anti-proliferative activity anthothecol. Therefore, the anti-proliferative activity of these four compounds on the growth of MCF7 cells was determined using the MTS assay (Table 11). In MCF-7 cells, all the compounds cause a 50% reduction in growth in the 0.5–10 µM range (Table 11). These results confirm the anti-proliferative properties of piplartine, rottlerin and garcinol, and establish anthothecol as anti-proliferative drug.

#### 3.2.3. Inhibition of HRI Maturation

**Figure 13:**
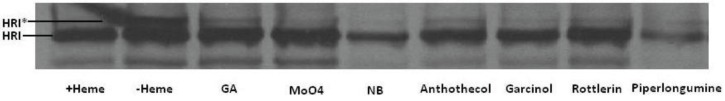
Effect of compounds on HRI’s Hsp90-dependent maturation. [^35^S]Labeled-HRI was synthesized by TnT in RRL and transferred to heme-deficient lysate for maturation. Translated protein was separated by SDS PAGE, transferred to PVDF membrane, and visualized by X-ray film exposure. The phosphorylated active form of the kinase is indicated with an asterisk. Lanes were treated as follows: heme, no heme, 20 µM geldanamycin (GA), 20 mM sodium molybdate, 20 mM novobiocin, and 100 µM each of anthothecol, garcinol, rottlerin, and piplartine/piperlongumine

The heme-regulated eIF2α kinase (HRI) is an Hsp90 client kinase which, upon folding by Hsp90, will activate, or mature, by autophosphorylation when heme is deficient [189]. This activation is dependent on functional Hsp90, and can be detected as an electophoretic mobility shift [189] when separated by polyacrylamide gel electrophoresis (PAGE) (Figure 13). Similar to the known Hsp90 inhibitors, geldanamycin (GA), molybdate and novobiocin, anthothecol, garcinol, rottlerin, and piplartine inhibited the maturation of HRI, as observed by the absence of the slower mobility form of HRI upon PAGE. This result further supports the hypothesis that physiological effects of these four compounds on cells are mediated, at least in part, through their ability to inhibit Hsp90.

#### 3.2.4. Compounds Induce Depletion of Hsp90-Dependent Clients

**Figure 14:**
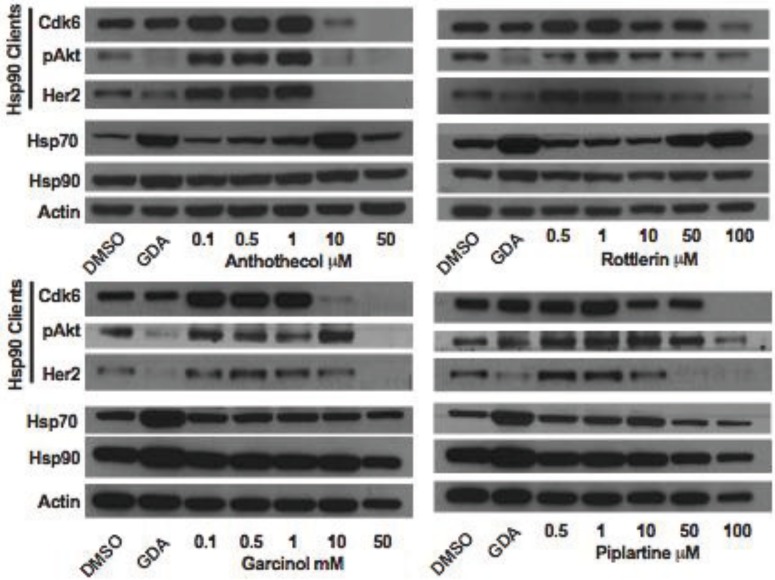
Western blot for Hsp90-dependent client proteins (Cdk2, pAkt and Her2), and Hsp90 and Hsp70 present in extracts prepared from MCF7 cells treated for 24 h with DMSO (vehicle control), 0.5 µM geldanamycin, or the indicated concentrations of anthothecol, rottlerin, garcinol, or piplartine. Actin was used as the loading control.

Inhibition of Hsp90 is well known to cause the depletion of Hsp90-depedent client proteins from inhibitor-treated cells. To further test the hypothesis that anthothecol, garcinol, rottlerin and piplartine are inhibitors of Hsp90, the effect of varying concentrations of the compounds on the expression of Hsp90-dependent client proteins Cdk6, pAkt, and Her2 in MCF7 cells was examined (Figure 14). Consistent with our hypothesis, all four compounds were observed to cause a concentration-dependent depletion of Hsp90 clients from MCF7 cells after 24 h of treatment. None of the compounds increased the expression of Hsp90. However, similar to geldanamycin, anthothecol and rottlerin caused a concentration-dependent increase in the expression of Hsp70, indicating that the compounds likely interact with the ATP binding site in Hsp90’s N-terminal domain. On the other hand, garcinol and piplartine had no effect on the expression of Hsp70, which is a property of compounds that inhibit Hsp90 by binding to its C-terminal domain [190].

## 4. Conclusions

The compounds identified from this screen represent diverse structures. Compounds with the greatest potential to be Hsp90 inhibitors are those that have already been identified as having specific activities, but against multiple targets. Often, reports by separate laboratories will demonstrate the activity of a compound against a certain kinase or pathway, while showing that related proteins or pathways are unaffected. When multiple targets with such effects are demonstrated, it is a strong indicator that the compound in question may be an Hsp90 inhibitor. Hsp90 is essential for the function of kinases, receptors, and other proteins from varied and wide-ranging pathways in the cell, but often plays no part in the function of closely related proteins. This scattered, yet specific, involvement of Hsp90 often precludes the detection of Hsp90’s importance by groups studying a narrow portion of the proteomic landscape. Accompanying the effects of these compounds is that they usually manifest anti-proliferative cytotoxic activities against cancer cells. Compounds that demonstrate these somewhat pleiotropic effects are amongst the first that should be considered for subsequent evaluations.

Another tell-tale sign of an Hsp90 inhibitor among screen hits is its anti-microbial activity. The earliest Hsp90 inhibitors, such as radicicol, geldanamycin, and novobiocin, were identified as antibiotics long before their activity against Hsp90 was elucidated.

It should be noted here that a portion of the compounds identified in the screening have structural properties that likely eliminate them from consideration for further characterization. Some compounds closely resemble compounds that are known intercalating agents. While these compounds could theoretically have some value as anti-cancer and anti-microbial drugs, we generally chose to bypass them, given their potential for off-target toxicity. Other compounds contain aldehyde moieties that can nonspecifically react with amino groups found in both proteins and nucleic acids. Another characteristic of compounds that potentially limit their usefulness is that some possess redox active quinones. While this may or may not affect their activity as Hsp90 inhibitors, the compounds can potentially induce oxidative stress, as well as chelate metal ions, and thus contribute to nonspecific effects in cells. An additional problem with a number of the compounds is that they contain α,β-unsaturated ketones, which can readily undergo Michael addition. 

Recent reviews have noted that aldehydes, quinones and α,β-unsaturated ketones are undesirable moieties and are generally avoided during drug development [191,192,193,194]. The presence of such moieties in small synthetic molecules often present in HTS libraries, has led to identification of a class of compounds that are pan-assay interference compounds (PAINS, [193,194]): compounds identified as hits in multiple screening assays due to their nonspecific biological activity. Indeed, it has been emphasized that development of compounds containing undesirable moieties as potential drug leads should proceed with caution, if they are to be pursued at all. In the field of development of Hsp90 inhibitors as potential chemotherapeutics this issue raises an interesting question in regards to the “poly”-pharmacological property expected from Hsp90 inhibitors: does the observed “poly”-pharmacology of a compound indicate that it is an Hsp90 inhibitor, or does it reflect the fact that it is a PAIN and is nonspecifically inhibiting multiple biological pathways in the cell? 

With regards to HTS, it should be noted that natural products are not indicative of the types of small synthetic molecules usually present in libraries utilized for HTS [191,195,196]. Natural products represent complex structures and, generally, a higher molecular weight. Natural products also occupy under-represented regions of biologically relevant chemical space: space that is often non-compliant with the rules of five [191,195,196]. Indeed, some drugs on the market based on natural products contain undesirable moieties [191]. In the context of the natural products presented herein, quinones are redox-active species that in some cases have been shown to exhibit preferential selectivity toward cancer cells [197]. In addition, we note that electrophilic moieties such as those found in α,β-unsaturated ketones are reactive species and further modification or retardation of their activity could be beneficial. However, it should be noted that a small set of compounds exhibiting such properties have advanced through clinical trials and have gained FDA approval [198,199]. 

The results presented here implicate the compounds anthothecol, garcinol, rottlerin, and piplartine/piperlongumine as inhibitors of the Hsp90 chaperone complex. Further study of the compounds will be necessary to confirm their status as inhibitors and Hsp90 as their target [200]. Similar studies will also be required to confirm or disprove whether the other compounds identified in the screening are inhibitors of the Hsp90 chaperone machine. The evidence presented for the +100 compounds being inhibitors of the Hsp90 chaperone machine obviously requires further support, and in no manner do we wish to imply that the compounds represent good lead compounds for therapeutic development. Instead, we offer this screening and literature mining as a resource for investigators interested in the possible mechanism of action of these natural compounds.
